# Editorial: Gene Therapy in the CNS – Progress and Prospects for Novel Therapies

**DOI:** 10.3389/fnmol.2021.778134

**Published:** 2021-10-20

**Authors:** Marco Ledri, Andreas T. Sørensen, Merab Kokaia, David P. D. Woldbye, Casper R. Gøtzsche

**Affiliations:** ^1^Laboratory of Molecular Neurophysiology and Epilepsy, Department of Clinical Sciences, Epilepsy Center, Faculty of Medicine, Lund University, Lund, Sweden; ^2^Department of Neuroscience, University of Copenhagen, Copenhagen, Denmark; ^3^Experimental Epilepsy Group, Department of Clinical Sciences, Epilepsy Center, Faculty of Medicine, Lund University, Lund, Sweden

**Keywords:** gene therapy, central nervous system, proof-of-concept, adeno-associated virus vector, genome editing, CRISPR

Gene therapy for central nervous system (CNS) diseases holds a compelling potential for the development of novel therapies. It offers the prospective of transformative and disease-modulating treatment opportunities with potential long-lived therapeutic effects. Recent advances in the field have renewed the optimism for the possibility to develop new innovative solutions and especially certain CNS diseases could benefit greatly from novel gene therapies.

Gene therapy encompasses the administration of biological medicinal products containing recombinant nucleic acids, administered to a human to regulate, repair, replace, add, or delete a genetic sequence with the aim to treat or cure diseases^1^. This includes *in vivo* vector-mediated gene therapy, *ex vivo* cell transduction gene therapy, and genome editing (Brenner et al., 2020). In the previous decade the first gene therapies were approved in Europe and/or USA, including Glybera^®^ (alipogene tiparvovec) for lipoprotein lipase deficiency (Watanabe et al., 2015), Strimvelis^®^ (*ex vivo* hematopoietic stem and progenitor cell (HSPC) gene therapy) for adenosine deaminase deficiency-induced severe combined immunodeficiency (ADA-SCID) (Aiuti et al., 2017), Zynteglo^®^ for β-thalassemia (Schuessler-Lenz et al., 2020), Luxturna^®^ (voretigene neparvovec) for inherited retinal dystrophy (Gao et al., 2020), Zolgensma^®^ (onasemnogene abeparvovec) for spinal muscular atrophy (Keeler and Flotte, 2019), and Libmeldy^®^ (*ex vivo* HSPC gene therapy) for metachromatic leukodystrophy (Bulaklak and Gersbach, 2020). These successes could be the beginning of the discovery, development, and approval of many new gene therapies in the near future. To realize new CNS gene therapies, multiple challenges must be addressed including identifying beneficial therapeutic targets, developing efficient administration and distribution techniques, documenting sustained treatment responses and long-term safety aspects, and demonstrating proof-of-concept for clinical improvements over current available standard of care.

This Research Topic aims at collecting articles describing novel discoveries and technologies relevant for development of gene therapies targeting CNS diseases including neurological, neurodevelopmental, neuroimmunological, neurodegenerative, neuro-oncological, and neuromuscular disorders. We here present 11 unique articles covering a broad span of original research and scientific field reviews within these topics.

First, Jensen et al. provide an overview of rare genetic diseases in the brain and spinal cord, where gene therapy is being investigated as new viable treatment strategies. The review describes the major progress at both the preclinical and clinical levels within degenerative, developmental, lysosomal storage, and metabolic disorders. This field has reached unprecedented milestones with recent market approvals by the FDA and EMA, and here the authors provide an overview of what could be the next breakthrough therapies.

Belur et al. demonstrate how a vector based upon adeno-associated virus (AAV) of serotype 9 was developed for alpha-L-iduronidase (IDUA) gene delivery by different routes of administration in a mouse model of mucopolysaccharidosis type I. The vector-induced gene expression was shown to increase the IDUA enzyme levels, leading to normalization of glycosaminoglycan levels and restored cognitive performance in a spatial memory model.

Cattaneo et al. summarize available preclinical data on neuropeptide Y (NPY) gene therapy for treatment of epilepsy, and discuss the anti-epileptic effects and critical aspects still remaining to be thoroughly investigated before clinical testing.

Szczygiel et al. demonstrate how AAV vector-mediated combinatorial delivery of NPY and its antiepileptic receptor Y2 unilaterally into the hippocampus of adult rats provides sustainable and neuron-specific transgene expression as long as 6 months post-injection. No significant side effects were observed on body weight or memory performance.

Audouard et al. investigate the short-term effects of using a novel AAV serotype, AAVPHP.eB, to introduce the gene expression of human lysosomal enzyme arylsulfatase A (hARSA) in a mouse model of metachromatic leukodystrophy (MLD). Three months after treatment, brain and spinal cord sulfatide storage was significantly decreased, and improvement of astrogliosis and microgliosis in brain and spinal cord was evident. These results support the AAVPHP.eB-hARSA gene therapy to be further tested in symptomatic rapidly progressing forms of MLD.

Banerjee et al. provide an overview of current approaches for glioma gene therapy and virotherapy, highlighting the progress, prospects, and challenges. Even though we still remain to see clinical success with innovative gene-mediated therapies and oncolytic virotherapies, the implementation of better preclinical translational models holds the potential for clinical breakthroughs in coming years.

Lubroth et al. present and discuss the current progress within *in vivo* genome editing and modifying technologies, including the clustered regularly interspaced short palindromic repeats (CRISPR) systems, zinc finger nucleases (ZFN), and transcription activator-like effector nucleases (TALEN), for translational neuroscience research and development of new treatments of CNS disorders. They also discuss the technical and commercial limitations as well as potential solutions to overcome these hurdles.

Rittiner et al. focus on the latest innovative solutions with delivery of therapeutic cargo to the nervous system using lentiviral and AAV vectors, overcoming problems associated with repeated drug administration and difficulties in delivering drugs across the blood-brain barrier. Centrally, they also describe how this technology can be applied in genome and epigenome-editing tools including CRISPR/Cas9 and the development of novel treatment of neurodegenerative diseases.

Zhu et al. describe how recent advances in gene sequencing and gene editing tools can be utilized for development of new therapies targeting neurodegenerative diseases. Not only is it possible to use gene editing for therapeutic approaches, it is also a valuable tool in research and to develop new experimental *in vitro* and *in vivo* disease models. Here the focus is on the progresses made in areas of Alzheimer's disease, Parkinson's disease, Huntington's disease, and amyotrophic lateral sclerosis.

O'Carroll et al. provide an overview of the potential and challenges for glial specific gene therapy, since different glial cell types are involved in nervous system pathology, playing roles in neurodegenerative disease and following trauma in the brain and spinal cord (astrocytes, microglia, oligodendrocytes), nerve degeneration and development of pain in peripheral nerves (Schwann cells, satellite cells), retinal diseases (Müller glia), and gut dysbiosis (enteric glia).

Finally, Tosolini and Sleigh outline how gene therapy can be administered with minimal invasiveness into skeletal muscles for extensive transduction of cells within the spinal cord, brainstem, and sensory ganglia, for treatment of neuronal conditions. In addition, they discuss optimization opportunities to the intramuscular administration route for improved gene delivery and therapeutic potential.

Taken together, the articles presented in this special issue of Frontiers in Molecular Neuroscience give a comprehensive overview of several important disciplines in gene therapy and provide novel insights into what could become viable treatments for CNS diseases.

## Author Contributions

CG drafted the manuscript. All authors contributed to the editing and finalization of the manuscript.

## Conflict of Interest

MK and DW are co-founders and consultants of CombiGene AB (Lund, Sweden), AS is founder of DolorestBio ApS (Copenhagen, Denmark), and CG is employed by UCB Nordic A/S (Copenhagen, Denmark). The remaining author declares that the research was conducted in the absence of any commercial or financial relationships that could be construed as a potential conflict of interest.

## Publisher's Note

All claims expressed in this article are solely those of the authors and do not necessarily represent those of their affiliated organizations, or those of the publisher, the editors and the reviewers. Any product that may be evaluated in this article, or claim that may be made by its manufacturer, is not guaranteed or endorsed by the publisher.

## Footnotes

^1^Based upon the definitions from the FDA (*Cellular & Gene Therapy Guidances, July 20, 2018*) and the EU commission (*Directive 2001/83/EC, Part IV of Annex I*).
